# The application of statistical methods to cognate docking: A path forward?

**DOI:** 10.1186/1758-2946-6-S1-P59

**Published:** 2014-03-11

**Authors:** Gunther Stahl, Paul CD Hawkins, Mark McGann, Matthew T Geballe, Gregory L Warren

**Affiliations:** 1 OpenEye Scientific Software, Cologne, 50672, Germany; 2 OpenEye Scientific Software, 9 Bisbee Court, Suite D, Santa Fe, NM, 87508, USA

## 

Cognate docking has been used as a test for pose prediction quality in docking engines for decades. While cognate docking is not the problem that docking engines are put to in their normal use (that being cross docking), it is expected that good performance in cognate docking is a necessary but not sufficient condition for good performance in cross docking. In this talk we report a statistically rigorous analysis of cognate docking using tools in the OEDocking suite [1,2]. We address a number of critically important aspects of the cognate docking problem that are frequently poorly handled in publications in this area; dataset quality, methods of comparison of the docked pose to the ligand model pose and analysis of the results to determine if and by how much a given method is actually better than another.

The first problem is handled through the use of our recently published Iridium-HT dataset [3]. To overcome the second problem we use a variety of measures to compare a docked pose to the ligand model pose. In addressing the third problem we utilize a variety of statistical methods to determine whether, and by how much, changes in the scoring functions actually improve cognate docking performance; a major challenge in this area is the paired nature of the deviation data. We caution against the mechanical application of statistical tests, however, and advocate for searching for substantive and meaningful significance, as well as statistical significance.
